# SMAD4-independent activation of TGF-β signaling by MUC1 in a human pancreatic cancer cell line

**DOI:** 10.18632/oncotarget.23966

**Published:** 2018-01-05

**Authors:** Priyanka Grover, Sritama Nath, Monica D. Nye, Ru Zhou, Mohammad Ahmad, Pinku Mukherjee

**Affiliations:** ^1^ Department of Biological Sciences, University of North Carolina at Charlotte, Charlotte, North Carolina 28223-0001, USA

**Keywords:** apoptosis, epithelial-mesenchymal transition (EMT), transforming growth factor beta (TGF-β), pancreatic cancer, mucin 1 (MUC1)

## Abstract

Pancreatic Ductal Adenocarcinoma (PDA) has a mortality rate that nearly matches its incidence rate. Transforming Growth Factor Beta (TGF-β) is a cytokine with a dual role in tumor development switching from a tumor suppressor to a tumor promoter. There is limited knowledge of how TGF-β function switches during tumorigenesis. Mucin 1 (MUC1) is an aberrantly glycosylated, membrane-bound, glycoprotein that is overexpressed in >80% of PDA cases and is associated with poor prognosis. In PDA, MUC1 promotes tumor progression and metastasis via signaling through its cytoplasmic tail (MUC1-CT) and interacting with other oncogenic signaling molecules. We hypothesize that high levels of MUC1 in PDA may be partly responsible for the TGF-β functional switch during oncogenesis. We report that overexpression of MUC1 in BxPC3 human PDA cells (BxPC3.MUC1) enhances the induction of epithelial to mesenchymal transition leading to increased invasiveness in response to exogenous TGF-β1. Simultaneously, these cells resist TGF-β induced apoptosis by downregulating levels of cleaved caspases. We show that mutating the tyrosines in MUC1-CT to phenylalanine reverses the TGF-β induced invasiveness. This suggests that the tyrosine residues in MUC1-CT are required for TGF-β induced invasion. Some of these tyrosines are phosphorylated by the tyrosine kinase c-Src. Thus, treatment of BxPC3.MUC1 cells with a c-Src inhibitor (PP2) significantly reduces TGF-β induced invasiveness. Similar observations were confirmed in the Chinese hamster ovarian (CHO) cell line. Data strongly suggests that MUC1 may regulate TGF-β function in PDA cells and thus have potential clinical relevance in the use of TGF-β inhibitors in clinical trials.

## INTRODUCTION

Pancreatic Ductal Adenocarcinoma (PDA) is the fourth leading cause of cancer related deaths in the United States with a median survival rate of less than six months and a 5–year survival rate of a dismal 7% [1, 2]. By 2030, PDA is predicted to be the second leading cause of cancer-related deaths in the United States [3]. Its mortality rate nearly matches its incidence rate [4].

Transforming Growth Factor Beta (TGF-β) is a cytokine with a dichotomous role in oncogenesis. In normal tissue development and early oncogenesis, the TGF-β signaling complex is a cell cycle regulator and induces apoptosis. The canonical pathway of TGF-β signaling starts with binding of two TGF-β Receptor type II (TGF-βRII) to two TGF-β Receptor type I (TGF-βRI) to activate the SMAD pathway [5, 6]. The receptors dimerize, when the ligand binds, triggering the activation of TGF-βRI kinase activity and switching it to a docking site for SMAD proteins [7]. SMAD 2 and SMAD 3 are activated by the TGF-βRI [8]. Once phosphorylated by TGF-βRI, SMAD 2 and 3 dimerize forming the SMAD 2/3 complex [9]. The SMAD 2/3 dimer joins with SMAD 4, creating a heterohexameric complex [9]. The newly created complex translocates to the nucleus, allowing for the transcriptional regulation of target genes which regulate cellular processes, such as induction of apoptosis [10]. However, it has been shown that in a SMAD 4 null cell line SMAD2 and SMAD3 are still able to translocate to the nucleus [11]. SMAD 4 is often mutated or deleted in about 55% of PDA cases showcasing the importance of studying SMAD4 independent mechanisms of PDA development [12]. Loss of functional SMAD 4 in PDA interferes with the TGF-β/SMAD pathway leading to decreased growth inhibition [13].

In later stages of cancer, a switch occurs and the TGF-β signaling pathway becomes a tumor promoter, inducing invasion and metastasis. TGF-β1 stimulates Epithelial-to-Mesenchymal Transition (EMT) through the activation of the ERK pathway [14]. As reviewed in Kalluri et al, EMT is a biological process that transforms an epithelial cell to a mesenchymal cell phenotype, which can lead to resistance to apoptosis [15]. Increased migration and invasion of cancer cells has also been associated with EMT [16]. The TGF-β switch in function from a tumor suppressor, via apoptosis, to a tumor promoter, via EMT, is elusive but holds high importance in treatment refractory cancers like PDA [17]. The TGF-β ligand family consists of three different, highly homologous isoforms: TGF-β1, TGF-β2, and TGF-β3 [18–20]. The most abundant isoform is TGF-β1 [9]. TGF-β is considered an important target for cancer therapy, and there are multiple anti-TGF-β compounds in clinical trials [21].

Mucin-1 (MUC1), a transmembrane glycoprotein that plays a critical role in tumor progression and metastasis in PDA [22]. In normal epithelial cells lining the ducts, MUC1 is localized on the apical surface and provides a protective barrier. However, when normal cells transform to malignant cells and lose their polarity, MUC1 is no longer restricted to the apical surface; it becomes hypo glycosylated, and comes in close proximity to several growth factor receptors including TGF-β receptors [23]. The tumor-associated form of MUC1 plays an important role in oncogenic signaling [24–27]. Studies have linked overexpression of MUC1 in tumors with enhanced EMT leading to increased invasiveness, metastasis, and drug resistance [22, 28, 29]. MUC1 induces increased production of prostaglandin (Cox-2) and growth factors (PDGF and VEGF), which leads to enhanced invasiveness of cells mainly through induction of EMT related genes [24, 27, 30, 31]. Importantly, MUC1 is overexpressed and aberrantly glycosylated in over 80% of PDA cases [22, 24, 30, 32, 33]. It is well established that the oncogenic signal transduction occurs through the cytoplasmic tail of MUC1 (MUC1-CT) [34, 35]. Once the MUC1-CT is phosphorylated, it associates with β-catenin and other transcription factors, and becomes released from the N-terminus of MUC1, leading it to translocate to the nucleus and subsequently activate downstream signaling pathways [25, 26, 36]. MUC1-CT is 72 amino acids long and is highly conserved with seven tyrosine residues that are phosphorylated by intracellular kinases. The phosphotyrosine residues act as a binding sites for molecules, such as c-Src, a proto-oncogene linked to cancer progression [22, 37].

In this study, we show that overexpression of MUC1 in human SMAD4 deleted PDA cell line BxPC3, plays an important role in the switch of TGF-β from a tumor suppressor to a tumor promoter, via a SMAD4 independent mechanism. Similar data is also reported in CHO cells. This study is the first to show that overexpression of MUC1 directly reduces TGF-β induced apoptosis and increases invasive potential in BxPC3 and CHO cells via signaling through the tyrosines in MUC1 CT.

## RESULTS

### Overexpression of MUC1 in BxPC3 and CHO cells significantly increases the amount of TGF-β1 produced without altering levels of the TGF-β receptors or SMAD2/3

**Figure 1:**
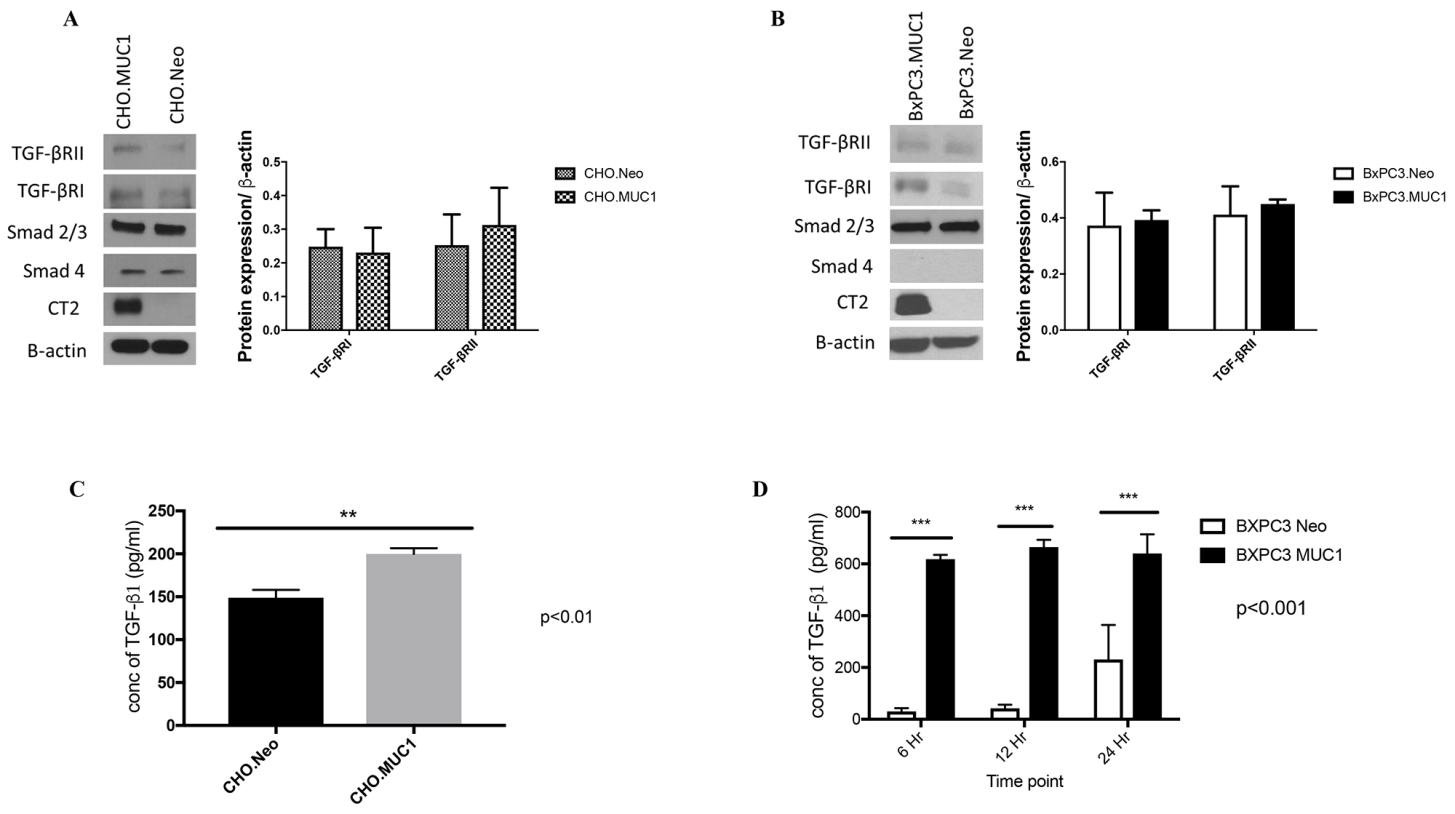
MUC1 overexpressing cells release significantly higher amounts of active TGF-β1 when compared to MUC1-low expressing cells **(A and B)** Western blotting detecting expression of MUC1-CT, TGF-βRI, TGF-βRII, SMAD 2/3, and SMAD4 in CHO and BxPC3 cells. Corresponding densitometric analysis for the TGF-β receptors. **(C and D)** TFG-β specific ELISA of supernatants from CHO and BxPC3 cells cultured in serum free medium for the indicated times. Results are presented as means +/− SEM of n=3. ^**^ p < 0.01, ^***^ p<0.001.

For this study, we selected Chinese hamster ovarian cell line (CHO) that is null for human MUC1 and a human PDA cell line BxPC3 that express low levels of endogenous human MUC1 and has SMAD4 independent TGF-β signaling, CHO cells have intact canonical TGF-β signaling pathway and were selected as a control cell line to investigate the effects of MUC1 on TGF-β signaling and phenotypic outcomes. Using a retroviral gene delivery system, we overexpressed the full-length human MUC1 transgene in BxPC3 and CHO cells creating two MUC1 high cell lines: BxPC3.MUC1 and CHO.MUC1. An empty vector, which does not carry the human MUC1 gene, was used to create the control cell lines BxPC3.Neo and CHO.Neo. Western blotting was performed to confirm the expression of human MUC1 in these cell lines. Cell lysates probed with CT2 antibody that recognizes the last 17 amino acids (SSLSYNTPAVAATSANL) of the cytoplasmic tail (CT) [38] revealed that BxPC3.MUC1 and CHO.MUC1 cells expressed high levels of human MUC1, while BxPC3.Neo and CHO.Neo did not (Figure 1A and 1B). Next, we tested expression of the key signaling components of the canonical TGF-β pathway, TGF-βRI, TGF-βRII, SMAD 2/3, and SMAD4 (expressed in CHO cells) [39]. We found that the levels of these signaling proteins were not significantly altered in the BxPC3.MUC1 compared to BxPC.Neo (Figure 1B) or in CHO.MUC1 compared to CHO.Neo (Figure 1A). Densitometric arbitrary units are shown in Figure 1A and 1B representing the levels of protein normalized to their β-actin loading control.

To investigate if overexpression of MUC1 alters SMAD4 independent TGF-β signaling, we first looked for differences in TGF-β1 secretion by these cells. Specific ELISA was used to determine the TGF-β1 concentration in the supernatant of these cells. Our data showed significantly higher levels of TGF-β1 in the supernatants of CHO.MUC1 at 48 hours and BxPC3.MUC1 at 6, 12, and 24 hours when compared to the control cell lines that expressed low levels of endogenous MUC1 (Figure 1C, p<0.01 and 1D, p<0.001), suggesting that MUC1 is a major contributor to the abundant release of TGF-β1. (Note: Only 48h time point is shown for CHO cells as earlier time points had very low undetectable levels of TGF-β1 release). Thus, we concluded that MUC1 overexpression increases TGF-β1 released but does not affect the expression of the receptors or the downstream signaling component.

### Overexpression of MUC1 protects PDA cells from TGF-β1-mediated apoptosis

**Figure 2:**
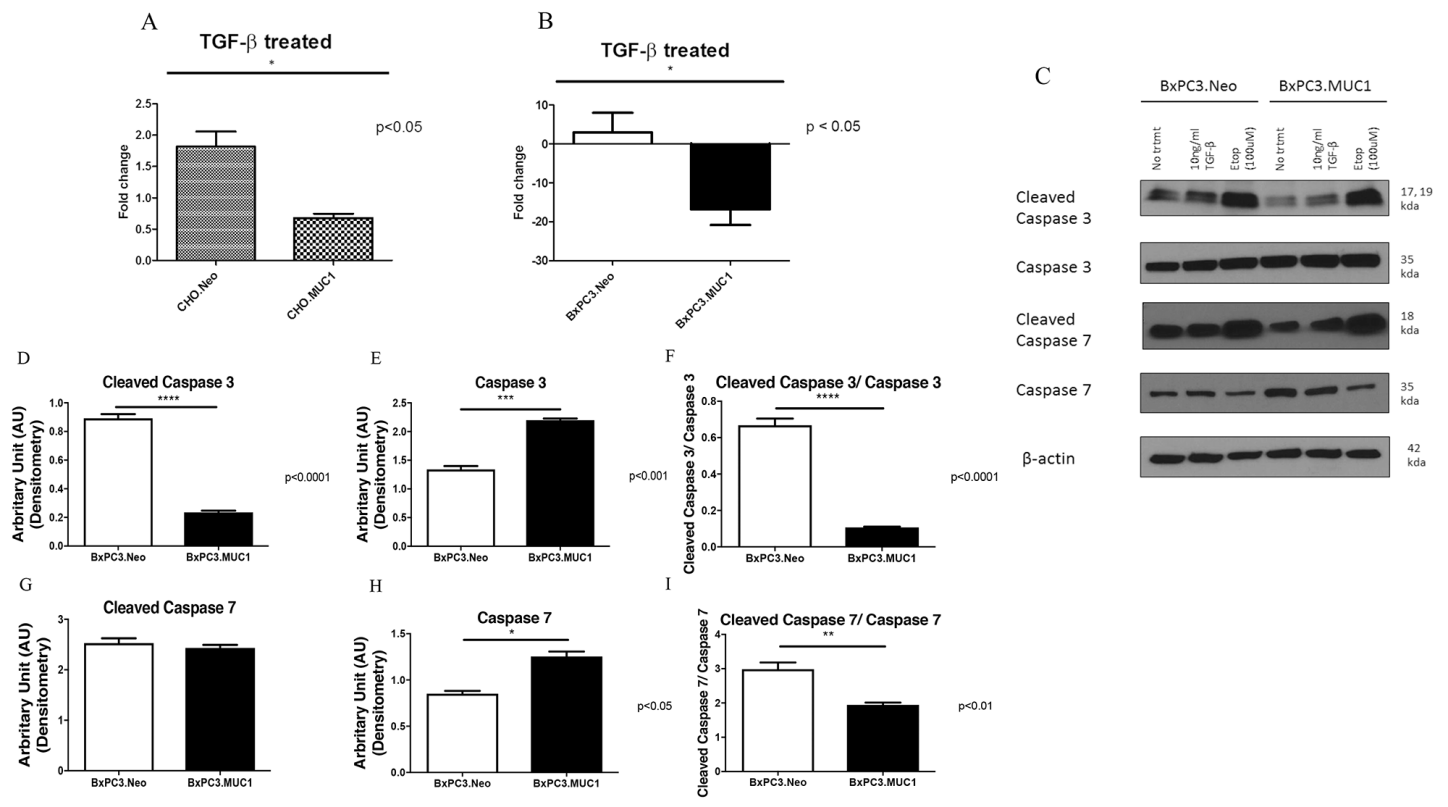
MUC1 overexpressing cells resist apoptosis in response to treatment with TGF-β1 with corresponding decrease in cleaved caspase 3 when compared to MUC1 low expressing cells **(A and B)** Apoptosis was determined at 48 hours post treatment with TGF-β1 by Annexin V+/7AAD staining and flow cytometry. Data is presented as fold change in apoptosis from untreated cells. **(C)** Western blotting of apoptotic markers (cleaved Caspase 3 and 7) in BxPC3 cells 48 hours post TGF-β1 treatment. **(D-I)** Corresponding densitometric analysis of C is presented. (D and G) Arbitrary densitometric unit of cleaved caspase 3 and cleaved caspase 7 normalized to β-actin respectively; (E and H) Arbitrary densitometric unit of total caspase 3 and caspase 7 normalized to β-actin; 2F: Ratio of cleaved caspase 3 and 7 normalized to total caspase 3 and 7. Results are presented as means +/− SEM of n=3. ^*^ p < 0.05, ^**^ p < 0.01, ^***^ p<0.001, ^****^ p<0.0001.

We determined the effect of exogenous TGF-β1 on induction of apoptosis in CHO and BxPC3 cells in context of MUC1 expression. Apoptosis was measured by performing Annexin V/7AAD staining followed by flow cytometry. Treatment with TGF-β1 induced a 2-fold induction of apoptosis in the CHO.Neo cells compared to 0.5-fold induction of apoptosis in CHO.MUC1 cells (Figure 2A, p<0.05). Similarly, BxPC3.MUC1 cells were completely protected from TGF-β1 induced apoptosis compared to 5-fold induction of apoptosis in BxPC3.Neo cells (Figure 2B, p<0.05). Furthermore, we found that TGF-β1 treatment activated cleavage of Caspase 3 more in the BxPC3.Neo cells than in the BxPC3.MUC1 cells (Figure 2C and 2D, p<0.0001) even though total Caspase 3 was significantly higher in the BxPC3.MUC1 versus the Neo cells (Figure 2C and 2E, p<0.001). Caspase 3 is a death protease commonly associated with changes in cell morphology, and induction of apoptosis [40]. MUC1 expression has been shown to reduce stress induced apoptosis by blocking activation of Caspase 8, which is known to interact and activate Caspase 3 [41]. It has also been shown to inhibit apoptosis under genotoxic stress via JNK1 activation [29, 42]. Upon comparing overall Caspase 3 activation, we observed that BxPC3.Neo has a statistically significant increase when compared to BxPC3.MUC1 in the presence of TGF-β1 (Figure 2C and 2F, p<0.0001). We did not observe any significant difference in cleaved Caspase 7 between BxPC3.Neo and MUC1 cells in response to TGF-β1 treatment (Figure 2C and 2G). However, when we compared the ratio of cleaved Caspase 7 versus total Caspase 7, a significant decrease in cleaved Caspase 7 in the MUC1-overexpressing cells was noted when exposed to TGF-β1 (Figure 2I, p<0.01). As with Caspase 3, Caspase 7 levels were significantly higher in BxPC3.MUC1 when compared to BxPC3.Neo cells (Figure 2C and 2H, p<0.05). Etoposide was used as the positive control for inducing Caspase 3 and 7 cleavage and activation. However, we did not observe any significant difference in Caspase 3 and 7 cleavages, because both BxPC3.MUC1 and BxPC3.Neo cells were equally sensitive to high concentration (100uM) of etoposide. Therefore, we suggest that cleaved caspases may regulate TGF-β induced apoptosis in the absence of MUC1. The densitometric arbitrary unit shown in Figures 2D, 2E, 2G, and 2H represent levels of protein normalized to their β-actin loading control while F and I represent levels of cleaved caspase/total caspase.

### Treatment with TGF-β1 increases invasive properties of MUC1-overexpressing cells as compared to their Neo counterparts

**Figure 3:**
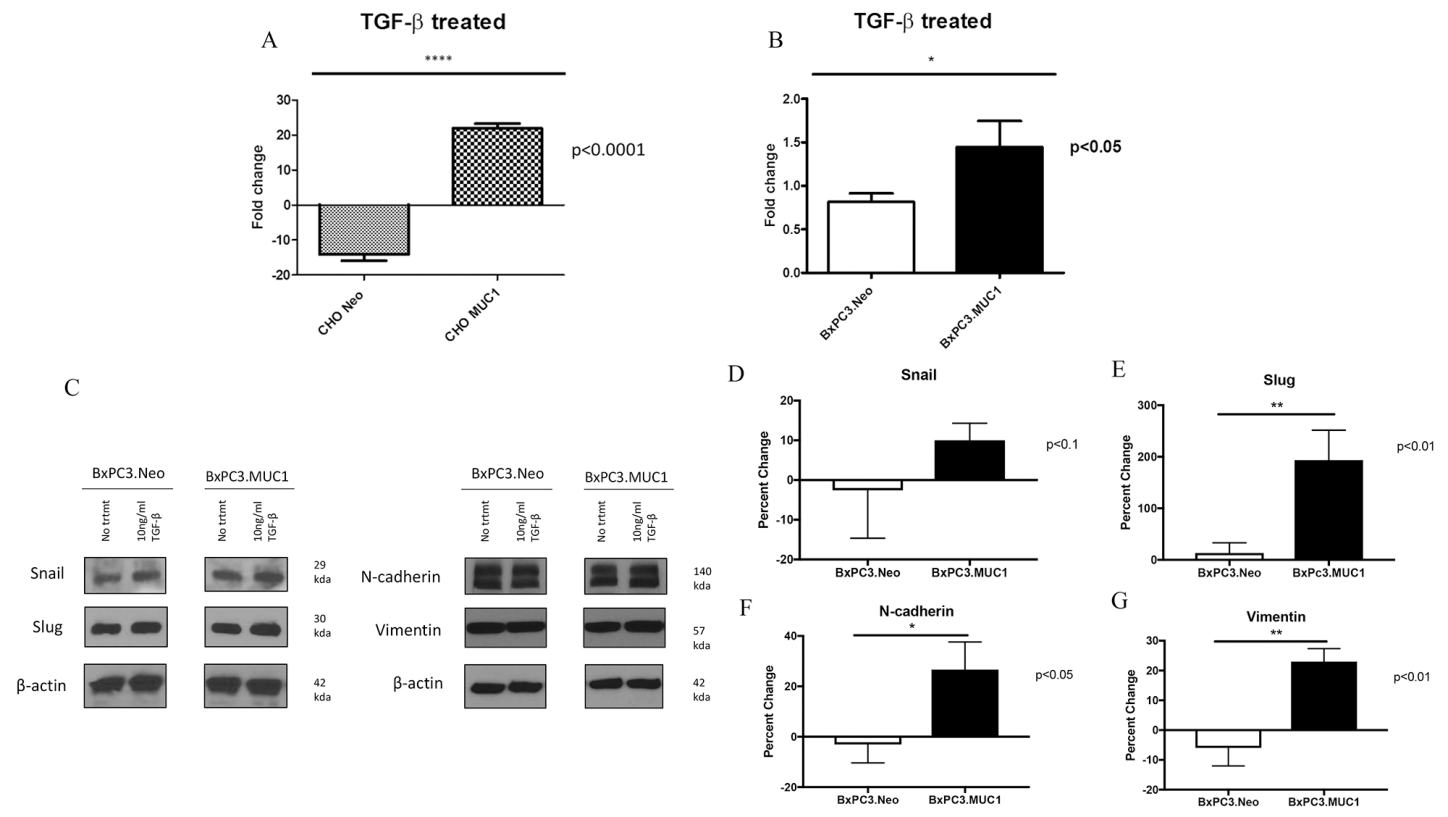
MUC1 overexpressing cells undergo significantly higher levels of invasion in response to TGF-β1 treatment **(A and B)** Invasion was determined by standard transwell assay at 48h time point. Results are presented as fold change from untreated. **(C)** Western blots to detect EMT markers 48 hours post TGF-β1 treatment. **(D-G)** Corresponding densitometric analysis of C is presented. Percent change from untreated is presented. All values are first normalized to its corresponding β-actin levels. Results are presented as means +/− SEM of n=3. ^*^ p < 0.05, ^**^ p < 0.01, ^****^ p<0.0001. D-G calculation: First the density value of each protein was normalized to their respective β-actin density value. Next the percent change was calculated by the formula: (TGF-β treated – No treatment/No treatment) ^*^ 100. If the final answer was negative, this was percentage decreased.

We hypothesized that TGF-β1 may induce invasiveness in MUC1-high but not MUC1-low cells by activating EMT. To test this hypothesis, we determined the invasive properties of BxPC3.MUC1 and CHO.MUC1 versus BxPC3.Neo and CHO.Neo cells in response to TGF-β1. Results show 20-fold higher levels of invasion in CHO.MUC1 when compared to CHO.Neo (Figure 3A, p<0.0001) and 1.5-fold higher in BxPC3.MUC1 when compared to BxPC3.Neo (Figure 3B, p<0.05). We recognize that CHO cells that are SMAD4 positive respond better to TGF-β. However, to further explore whether SMAD4 deletion plays a role, we also tested the invasive potential of Wild Type SMAD4 PDA cell lines HPAF-II and MIA PaCa-2 (Supplementary Figure 4). HPAF-II, an endogenously high MUC1 line significantly increases its invasive potential when exposed to TGF- β1. Following the trends established, MIA PaCa-2, an endogenously low MUC1 line, significantly decreases its invasive potential in the presence of TGF- β1. These cell lines, in relation to their endogenous MUC1 levels, will be further studied. Overall, the results suggest that there is synergistic interaction between MUC1 and TGF-β signaling resulting in increased motility and invasiveness. Next, we assessed the levels of EMT associated proteins by western blotting in TGF-β1 treated versus untreated cells. Forty-eight hours post TGF-β1 treatment, levels of Snail, Slug, Vimentin, and N-Cadherin was determined. The percent change in density of the bands due to TGF-β1 treatment is significantly higher in the BxPC3.MUC1 compared to BxPC3.Neo for all the EMT associated proteins except for Snail (Figure 3C-3G). Percent change was determined by formula (TGF-β treated – No treatment/No treatment) ^*^ 100. If the final answer was negative, this was percentage decrease (suggesting that the protein level remained unchanged with treatment). We observed no difference in the activation of the ERK pathway when examining levels of phospho-ERK between MUC1 and Neo cells. Presently, we do not know why that is, however we suspect that in the absence of SMAD4 in the MUC1 overexpessing BxPC3 cell line that the ERK pathway may not be activated [43].

### TGF-β mediated functions require signaling through the tyrosines present in MUC1-CT

**Figure 4:**
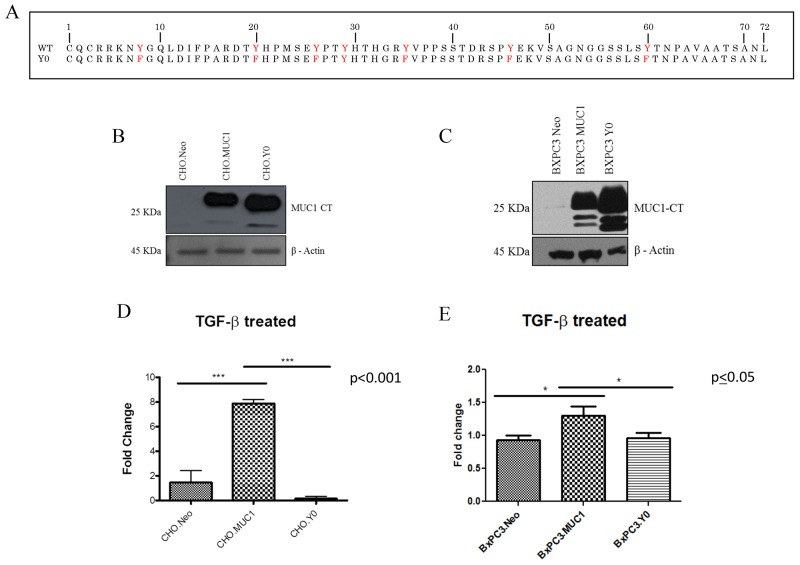
TGF-β1 mediated invasiveness is dependent upon signaling through the tyrosines in MUC1-CT **(A)** Amino Acid sequence of MUC1 CT WT and MUC1 CT Y0 where tyrosines are mutated to phenylalanine. **(B and C)** Western blots to detect MUC1 using the MUC1-CT antibody in CHO.Neo, MUC1, and Y0 cells as well as BxPC3.Neo, MUC1, and Y0 cells. **(D and E)** 48 hour invasion in response to TGF-β1 treatment presented as fold change from untreated cells. Results are presented as means +/− SEM of n=3. ^*^ p<0.05, ^***^ p<0.001.

We next investigated if the functional differences of TGF-β were manifestations of signaling crosstalk between the TGF-β signaling components and MUC1-CT. MUC1 associated non-canonical regulation of TGF-β signaling in a SMAD4 independent mechanism is responsible for the activation of other transcription factors via their interaction with the cytoplasmic tail of MUC1 [44]. Therefore, we hypothesized that the interaction of MUC1-CT with the TGF-β signaling pathway regulates the differences in apoptosis and induction of EMT independently of SMAD4. To test this hypothesis we generated a phosphomutant form of MUC1 (CHO.Y0 and BxPC3.Y0), where all seven tyrosines of MUC1-CT were mutated to phenylalanine. The MUC1 Y0 mutant is considered ‘a non-functional form’ of MUC1 CT as it lacks the tyrosines for phosphorylation, a precursor for downstream signal transduction (Figure 4A). Western blots show the expression levels of MUC1-CT in Neo, MUC1, and Y0 cells (Figure 4B and 4C). As previously observed, TGF-β1 treatment increases invasiveness in the MUC1-overexpressing cells when compared to the Neo cells. However, when comparing phosphomutant BxPC3.Y0 or CHO.Y0 cells to the full-length MUC1-overexpressing cells, we observed a complete reversal of the enhanced invasion when exposed to TGF-β1 (Figure 4D and 4E). Since the only difference between the full length MUC1 and MUC1.Y0 expressing cells is the ability to signal through the tyrosine residues of MUC1-CT. We postulate that the tyrosine residues of MUC1-CT are critical for the synergistic cross talk between MUC1 and TGF-β signaling that results in the TGF-β associated apoptosis and invasion. To our surprise we observed an increase in Vimentin in the BxPC3.Y0 cells post TGF-β1 treatment (Supplementary Figure 1A); however, it was striking to note that there were no cleaved caspases 3 or 7 in these cells post TGF-β1 treatment suggesting that the tyrosines play a major role in the TGF-β induced apoptotic pathway (Supplementary Figure 1B). To confirm that treatment itself did not cause any changes in the MUC1 levels, we conducted western blotting for MUC1 extracellular domain expression pre and post-TGF-β1 or etoposide treatment in BxC3.Neo, MUC1, and Y0 cells (Supplementary Figure 2). Treatment did not result in any change in the expression levels of MUC1 in the cells. Due to the changes in tyrosine to phenylalanine, the Y0 cells always run smaller in size and has been published extensively [45].

### C-Src inhibition negates TGF-β mediated invasion in MUC1-overexpressing cells

**Figure 5:**
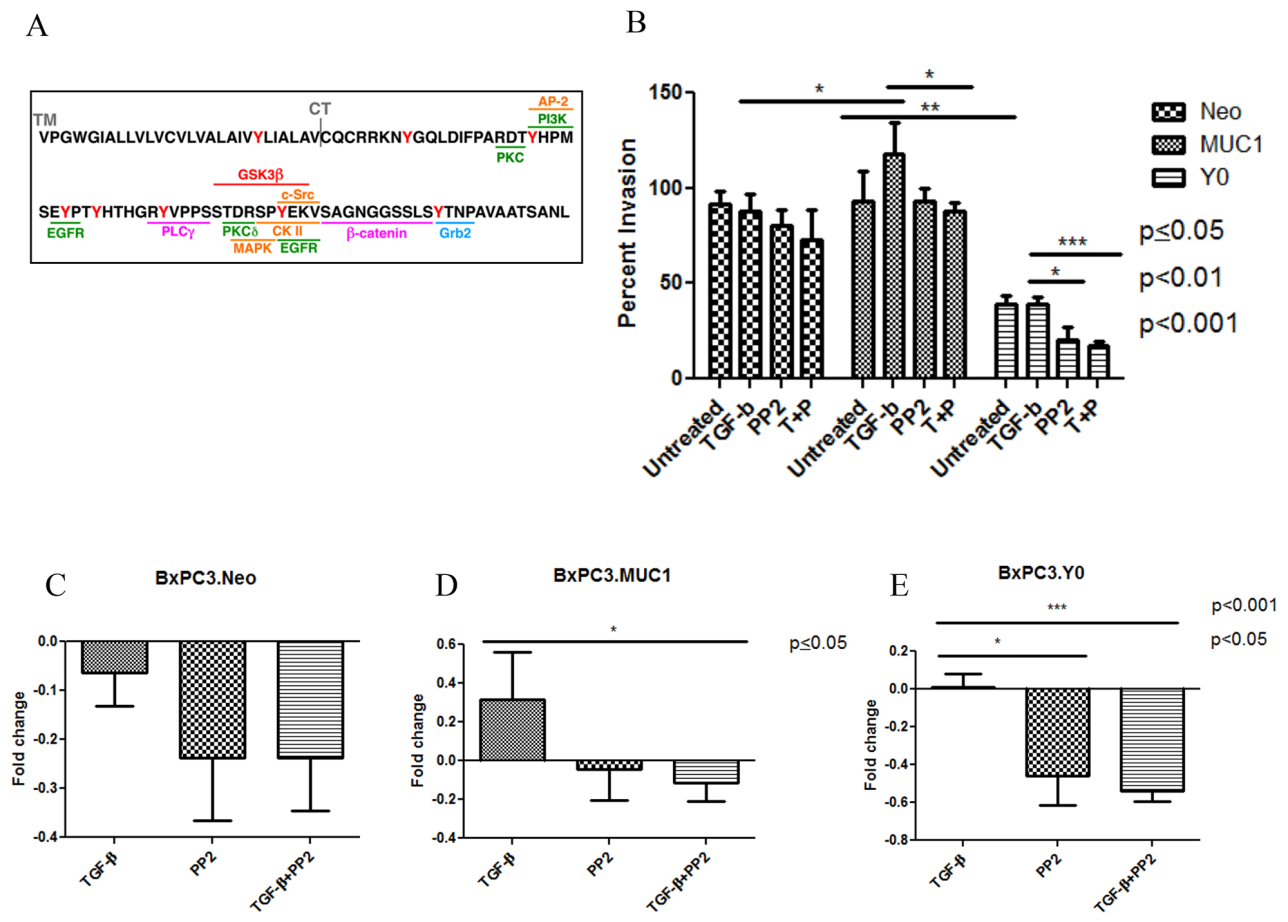
C-Src inhibition negates the aggressiveness of TGF-β mediated invasion in MUC1 expressing cells **(A)** Schematic of MUC-CT amino acid sequence and the potential kinase binding sites. **(B)** Percent invasion was determined by standard transwell assay at 48 hours post treatment with TGF-β1 ± PP2 as indicated in the figure. **(C-E)** Each treatment is compared to the untreated within each individual cell line. Results of the invasion assay are presented as means +/− SEM of n=3. ^*^ p<0.05, ^***^ p<0.001.

It has been shown that when Dasatinib, a Src inhibitor, was used on PDA cell lines PANC-1 and Colo-357, it inhibited TGF-β1 induced SMAD phosphorylation, migration, and invasion, therefore it is a tyrosine to consider [46]. c-Src is also associated with MUC1-CT and plays a vital role in MUC1 induced tumor metastasis [22, 37, 47]. Therefore, when BxPC3.MUC1 cells were treated with PP2, a c-Src inhibitor, the invasiveness of the cells in response to TGF-β1 was decreased (Figure 5B and 5D, p<0.05). However, PP2 treatment did not affect the invasive potential of BxPC3.Neo cells (Figure 5B and 5C). Although the BxPC3.Y0 cells had lower percent invasion than BxPC3.MUC1 and BxPC3.Neo cells, it is interesting that PP2 treatment further decreased invasiveness in BxPC3.Y0 cells (Figure 5B and 5E, p<0.001, p<0.05). The fact that PP2 inhibition affected the Y0 cells may be because PP2 is non-selective and is known to weakly inhibit EGFR and many other kinases with similar affinities [48, 49]. Overall, the results suggest that overexpression of MUC1 in these cell lines drive the anti-apoptotic oncogenic functions of TGF-β in a SMAD4 independent manner, and that this is partially via signaling interaction of c-Src with MUC1-CT. Further investigations need to be conducted to better understand the mechanisms and importance of MUC1-CT tyrosines and the interaction with other oncogenic signaling pathways. In a pilot study, we established the MUC1 CT expression levels and the natural invasive potential of a variety of MUC1-CT mutated BxPC3 cells (Supplementary Figure 3A and 3B). In BxPC3.Y2 and 5; BxPC3.Y6; BxPC3.Y7; and BxPC3.Y3, 6 and 7 cell lines where either single or multiple tyrosines are mutated to phelyalanine, the results show that these cells behave similarly to BxPC3.Y0. These results further establish the critical oncogenic role of MUC1 CT as reviewed in [44].

It must be noted that the levels that we report for the endogenous TGF-β1 production is in picograms/ml and what we add exogenously is in ngs/ml. In the CHO.MUC1 cells, the level is only 0.1ngs and in BxPC3.MUC1, it is 0.6ngs (Figure 1). Therefore, the functional differences we report in Figures 2-5 is due predominantly through the exogenous addition of TGF-β (10ngs).

## DISCUSSION

In a noncancerous pancreas, MUC1 is expressed in low levels on the luminal surface of the ductal epithelial cells. Yet, an exponential increase in MUC1 expression occurs during early stages of pancreatic cancer development. Even in early stage pancreatic intraepithelial neoplasia (PanIN) lesions, there is an observed increase in MUC1 expression [27, 50, 51]. It is also shown that TGF-β mediated response changes from apoptotic and cell growth regulatory to increasing invasiveness and migration in cancer [9]. The data presented herein suggests that MUC1's interaction with components of the TGF-β signaling pathway, in a SMAD4 independent mechanism, increases the oncogenic features of anti-apoptosis, increased EMT signaling, and more invasion. This has important clinical relevance, because MUC1 may be a biomarker for anti-TGF-β therapies in PDA cells. Tumors with high MUC1 expression can now be considered for TGF-β neutralizing strategies, while MUC1 low expressing tumors should not be considered for the same.

Using a SMAD4 independent PDA cell model, we demonstrate that MUC1 increases TGF-β1 secretion, without affecting expression of the key components of the TGF-β signaling in a SMAD-4 deleted cell line (Figure 1). We believe that the increase in TGF-β1 secretion in the MUC1 overexpressing cells (Figure 1) may be due to the 3-fold increase in latent TGF-beta binding protein 1 (LTBP-1) gene expression in the BxPC3.MUC1 when compared to the Neo cells (from our microarray data^1^ (data not shown)). LTBP-1 activates TGF-β secretion. This targets latent complexes of TGF-β to the extracellular matrix, where the latent cytokine is subsequently activated by various mechanisms. It has been previously shown that MUC1 expression increased TGF-β1 expression at the mRNA and protein levels in human hepatocellular carcinoma cells [52]. In dry eye disease, it has also been shown that MUC1 increases basal TGF-β expression [53]. Recently, it has been shown that TGF-β signaling and deletion of SMAD 4 can alter AGR2 expression, which in turn positively correlates with MUC1 expression [54]. These studies support our findings that in a MUC1-overexpressing SMAD 4 deleted PDA cell line model, MUC1 increases TGF-β1 expression and release. The mechanisms for upregulation of TGF-β1 are unknown. However, once malignant cells lose their growth inhibitory response to TGF-β1 and produce high levels of these protein, the increased expression of TGF-β1 provides a selective advantage for tumor cell survival as TGF-β1 are also angiogenic and have potent immunosuppressive effects [39].

During the early phases of tumorigenesis, TGF-β inhibits primary tumor development and growth by inducing cell cycle arrest and apoptosis [55, 56]. Apoptosis is characterized by morphological and biochemical changes [57]. When the role of TGF-β changes from tumor suppressor to tumor promoter, as reviewed in Lebrun 2012, the tumor promoting effects of TGF-β includes induction of EMT, resistance to apoptosis, migration, invasion, and tumor metastasis [58]. It has been shown that SMAD-4 deleted WT BxPC3 cells constitutively activates ERK, has an increased anti-apoptotic response but does not promote invasiveness [43, 59]. Finally, it has also been shown that MUC1 expression can confer resistance of epithelial cancer cells to cell death via anoikis [60]. Data from our study indicates that MUC1-overexpressing cells are resistant to TGF-β mediated apoptosis, (Figure 2) and become highly invasive in a SMAD4-independent manner (Figure 3). We have also shown similar results in an endogenously MUC1 high Wild Type SMAD4 PDA cell line (Supplementary Figure 4). Another study reported that inhibiting TGF-β downstream signaling reduces invasiveness in PANC-1 PDA cell line that is known to express MUC1 [61]. Thus, our data correlates with that study, showing that the MUC1-over expressing cell lines, BxPC3.MUC1 and CHO.MUC1, have significantly reduced TGF-β-induced invasiveness when downstream signaling is blocked in the MUC1 phosphomutant Y0 cells or in PP2 treated cells (Figures 4 and 5). The blocking of MUC1-CT downstream signaling in SMAD4 - negative pancreatic cancer cell line reduces the effects previously seen in the MUC1-high expressing cells, establishing the importance of MUC1-CT. This is significant for the 55% of PDA cases where SMAD4 is deleted. It is important to note that MUC1 expression level does not change with TGF-β1 treatment or in cells with MUC1 CT tyrosines mutated to phenylalanine (Supplementary Figure 2). Therefore, the effects are not a reflection of differences in MUC1 expression levels. Although MUC1 is known to confer resistance to apoptosis in response to several genotoxic drugs in PDA and other cancer cells [29, 41, 42], this is the first study that shows MUC1 blocks TGF-β induced apoptosis. Signaling through the CT of MUC1 is critical for cleavage of caspases and apoptosis (Supplementary Figure 1B).

**Figure 6:**
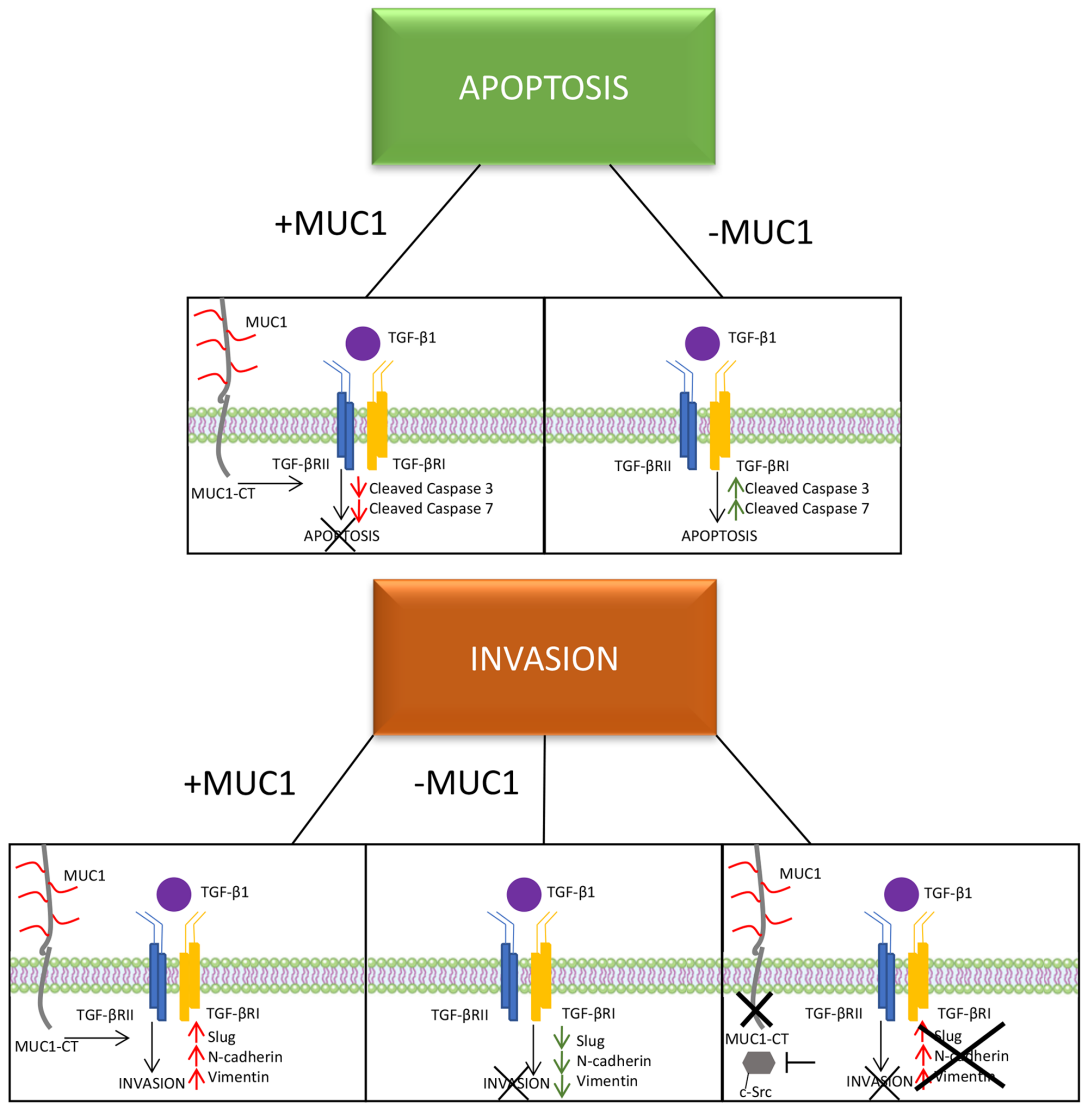
A schematic of the proposed mechanism of MUC1 mediating TGF-β signaling Schematic showing that MUC1-CT plays an important role in switching the role of TGF-β from a tumor suppressor to a tumor promoter in PDA, specifically in BxPC3 cells.

Previous studies have shown that the clinical efficiency of inhibition of c-Src in PDA cells is due to inhibition of tumor-promoting TGF-β signaling [46]. Our data supports this interaction by providing evidence that in BxPC3.MUC1 cells treated with c-Src inhibitor PP2 significantly reduced TGF-β-induced invasion (Figure 5). However, it is also shown that PP2 can be non-selective by weakly inhibit EGFR and have other off-target effects [48, 49]. Further array analysis can be performed to understand the complete mechanism. Solving the mystery of the molecular interactions with other oncogenic signaling pathways associated with SMAD4 independent TGF-β signaling will provide great insight into the functional switch of TGF-β from a tumor suppressor to a promotor of tumor development. This knowledge may potentially enable anti-TGF-β therapies in combination with standard therapies and/or immunotherapy to become more efficiently used in the clinic. For example, although certain TGF-β inhibitory treatments have worked *in vivo* using mouse models, the results have not been particularly promising in clinical trials [62]. Targeting TGF-β carries a substantial risk as this pathway is implicated in multiple homeostatic processes and is known to have tumor-suppressor functions. Establishing the mechanism, and determining a potential biomarker, should be priority before continuing anti-TGF-β clinical trials. The mechanisms for upregulation of TGF-β remain unknown. However, once malignant cells lose their growth inhibitory response to TGF-β and produce massive amounts of TGF-β (as seen in the MUC1-high cells-Figure 1), the increased expression of TGF-β provides a discerning advantage for tumor cell survival. If MUC1 can be shown as a correlative biomarker, as our data suggests, we may alleviate some of the complications associated with anti-TGF-β therapies, especially in SMAD4 independent PDA cases. The data presented here is just the beginning in establishing why certain patients may be more suitable candidates for TGF-β targeted therapies than others may. We conclude that signaling through MUC1-CT plays a critical role in the switch of SMAD4 independent TGF-β function from a pro-apoptotic to a pro-invasion cytokine (Figure 6).

## EXPERIMENTAL PROCEDURES

### Cell lines and culture

CHO.MUC1, CHO.Neo, CHO. Y0, BxPC3.MUC1, BxPC3.Neo, BxPC3.Y0 were generated as previously described [30]. HPAF-II and MIA Paca-2 were obtained from American Type Culture Collection and cultured as instructed. Cell lines were maintained in Roswell Park Memorial Institute 1640 medium (RPMI; with, L-glutamine; ThermoFisher). RPMI was supplemented with 10% fetal bovine serum (FBS; Hyclone), 3.4 mM L-glutamine, 90 units (U) per ml penicillin, 90 μg/ml streptomycin, and 1% Non-essential amino acids (Cellgro). RPMI was also supplemented with Geneticin (G418; Invitrogen, Carlsbad, CA, USA). Cells were kept in a 5% CO_2_ atmosphere at 37°C. The antibiotic G418 (50 mg/ml) was added to every passage of BxPC3.Neo and BxPC3.MUC1 to ensure positive selection of MUC1 positive cells. For all experiments, cell lines were passaged no more than 10 times.

### Western blotting

Cellular lysate preparation and Western blotting was done as previously described [30]. The cells were either treated as such: no treatment, 10 ng/ml of TGF-β1 (Peprotech, Rocky Hill, NJ, USA), or 100μM of Etoposide for 48 hours due to more pronounced signaling. 1:500 Armenian hamster monoclonal anti-human MUC1 cytoplasmic tail (CT2) antibody was used to probe for MUC1 in phosphate- buffered-saline-Tween 20 (PBS-T) with 5% BSA. CT2 antibody recognizes the last 17 amino acids (SSLSYNTPAVAATSANL) of the cytoplasmic tail (CT) of human MUC1 [38]. 1:10,000 TAB004 (OncoTAb, Charlotte, NC) was used to detect the N-terminus extracellular domain of MUC1 [24, 51]. Membranes were also probed with the following antibodies from Cell Signaling (1:1000): Smad4 (Rabbit, 38454), Smad 2/3 (Rabbit, 5678), Vimentin (Rabbit, 5741), Snail (Rabbit, 3879), Slug (Rabbit, 9585), N-cadherin (Rabbit, 13116), Cleaved Caspase 3 (Rabbit, 9664), Caspase 3 (Rabbit, 9665), Cleaved Caspase 7 (Rabbit, 8438), Caspase 7 (Rabbit, 12827), and β-Actin (Mouse, 3700). Other antibodies used include TGF-βRI (Abcam, 1:200, Rabbit, ab31013) and TGF-βRII (Abcam, 1:1000, Rabbit, ab61213). Densitometric analysis was conducted using the ImageJ software and percent change is calculated accordingly: First, each density unit for the particular protein was normalized to their respective β-actin density. Percent change was determined by formula (TGF-β treated – No treatment/No treatment) ^*^ 100. If the final answer was negative, this was percentage decrease (suggesting that the protein level remained unchanged with treatment).

### ELISA

Cells plated in duplicates in 6 well plates were cultured for 6, 12, and 24 hours. At the indicated time point, the culture supernatant was collected and concentrated using Amicon ultra-centrifugal filters (3KDa cutoff). The protein retenate was reconstituted up to 0.5ml with PBS. TGF-β1 levels in the supernatant were assessed using a specific ELISA (R&D systems, Minneapolis, MN), according to the manufacturer's recommended protocol. The total protein concentration was determined by BCA. The TGF-β1 levels were normalized to the total protein content of each sample. Results were expressed as TGF-β1 pg/ml concentration.

### Apoptosis assay

Cells that were serum starved for 18 hours were left untreated or treated with 10ng/ml of TGF-β1 (Peprotech, Rocky Hill, NJ, USA) and 100μM of Etoposide (as a positive control). 24 hours after treatment began; the cells were harvested and stained with Annexin V and PI (Annexin V/Dead Cell Apoptosis Kit, Life Technologies, Eugene, Oregon). The cells were analyzed using BD FORTESSA and FlowJo Version 8.8.7. Fold-change was calculated as TGF-β treated percent apoptosis/control percent invasion.

### Invasion assay

Cells were serum starved 18 hours before plating for the invasion assay. 50,000 cells in serum-free media were plated over transwell inserts (BD Biosciences, San Jose, CA, USA) precoated with diluted reduced growth factor Matrigel in serum free media, plus or minus TGF-β1 (Peprotech, Rocky Hill, NJ, USA). The cells were allowed to invade through the Matrigel for 48 hours towards the serum contained in the bottom chamber. After 48 hours, only the control wells were swabbed with a cotton swab, followed by staining of all inserts with coomassie blue. The excess stain was washed off and the inserts were allowed to dry. The membrane was cut and dipped in 10% acetic acid for 10 minutes to elute the dye, which was read by UV/Vis Spectrophotometer at 562μM. Percent invasion was calculated as sample absorbance/control absorbance X 100. Fold-change was calculated as TGF-β treated percent invasion/untreated percent invasion.

### Treatment with c-Src inhibitor

BxPC3.MUC1, Neo, and Y0 cells were serum starved 18 hours before plating for the invasion assay. 50,000 cells were plated as in the invasion assay protocol. Cells were either left untreated, treated with 10 ng/ml of TGF-β1 (Peprotech, Rocky Hill, NJ, USA), or the c-Src inhibitor PP2 (Tocris), or a combination of 10 ng/ml of TGF-β1 and PP2. The invasion assay was performed as described above.

### Statistics

Graphpad Prism 5 and ImageJ were used to analyze the western data. Graphpad Prism 5 was used to create the graphs and perform statistical analysis.

### Footnotes

The content is solely the responsibility of the authors and does not necessarily represent the official views of the National Institutes of Health.

## SUPPLEMENTARY MATERIALS FIGURES



## Abbreviations

PDA: Pancreatic Ductal Adenocarcioma
TGFβ: Transforming Growth Factor Beta
MUC1: Mucin 1
MUC1.CT: Mucin 1 cytoplasmic tail
BxPC3.MUC1: BxPC3 cell line overexpressing human MUC1 protein
PP2: c-Src inhibitor
CHO: Chinese Hamster Ovarian cell line
TGFβRI: Transforming Growth Factor Beta Receptor I
TGFβRII: Transforming Growth Factor Beta Receptor II
EMT: Epithelial- Mesenchymal Transition
COX-2: cyclooxygenase-2
PDGF: Platelet Derived Growth Factor
VEGF: Vascular Endothelial Growth Factor
CT: cytoplasmic tail
CHO.MUC1: Chinese Hamster Ovarian cell line overexpressing human MUC1 protein
BxPC3.Y0: BxPC3 cell line expressing Mucin 1 protein where all 7 tyrosines are mutated
CHO.Y0: Chinese Hamster Ovarian cell line expressing Mucin1 protein with 7 mutated tyrosines
Y0: mutated tyrosines
PanIN: Pancreatic intraepithelial lesions
LTBP1: Latent Transforming Growth Factor Beta Binding Protein.
