# Supplementary material for: SMAD4-independent activation of TGF-β signaling by MUC1 in a human pancreatic cancer cell line
Source: Oncotarget. 2018 Jan 5;9(6):6897–910. doi: 10.18632/oncotarget.23966 (PMC5805524; doi:10.18632/oncotarget.23966)
Supplement: Supplementary file 1 [file oncotarget-09-6897-s001.pdf]

SMAD4-independent activation of TGF-β signaling by MUC1 in a human pancreatic cancer cell line

SUPPLEMENTARY MATERIALS

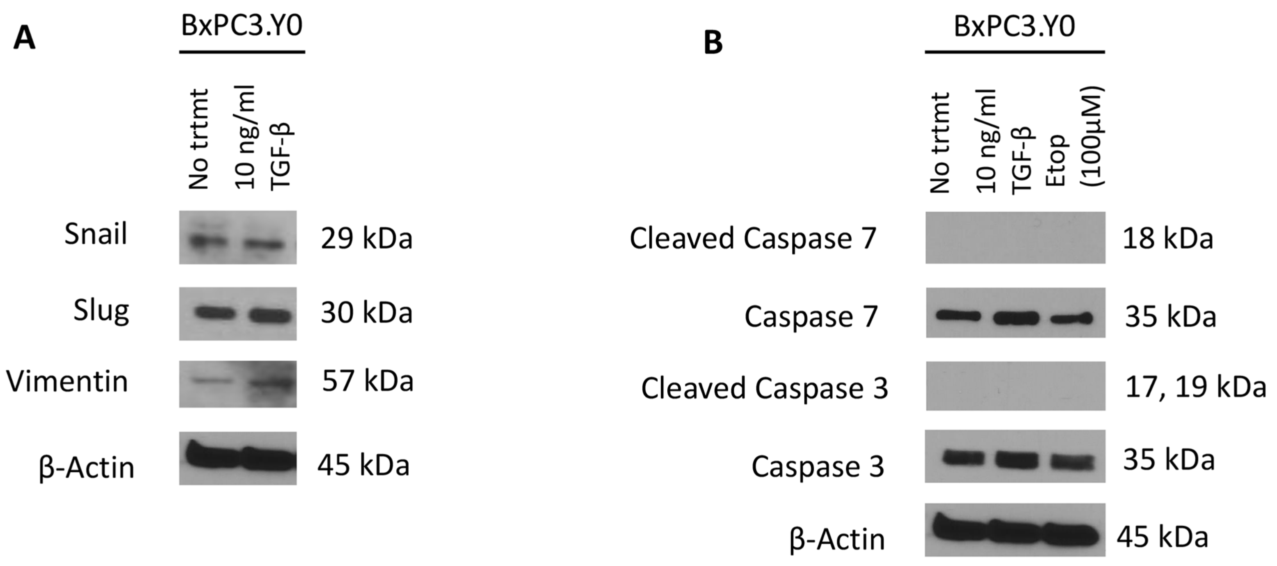

Supplementary Figure 1: Western blotting showing **(A)** EMT and **(B)** Apoptosis associated markers in BxPC3.Y0 cells in response to exogenous TGF- β1.

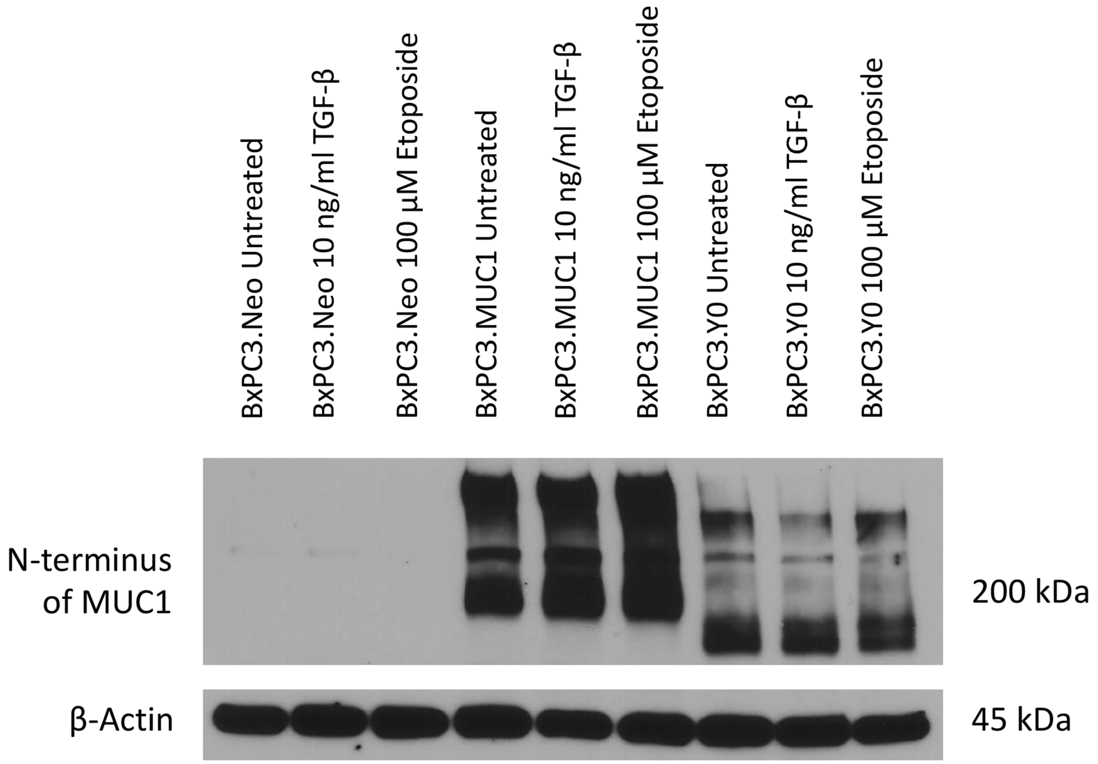

Supplementary Figure 2: N-terminus of MUC1 expression in BxPC3.Neo, BxPC3.MUC1, and BxPC3.Y0 under various conditions was analyzed by Western Blot. Due to the changes of tyrosine to phenylalanine, the Y0 cells always run smaller in size.

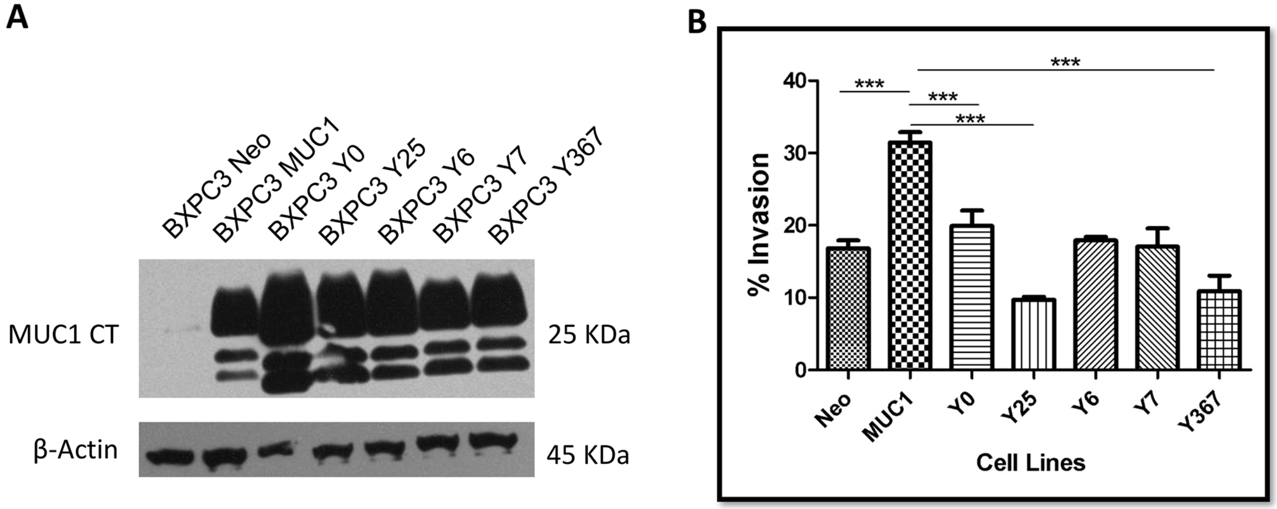

Supplementary Figure 3: (A) MUC1 expression in BXPC3 cells infected with full length MUC1 or various mutants of MUC1 CT was analyzed by Western Blot. **(B)** BXPC3 cells stably expressing the various mutants of MUC1 CT were plated over transwell inserts pre-coated with reduced growth factor matrigel, and were allowed to invade the matrix towards serum contained in the bottom chamber for 48 hours. Percent invasion was calculated as absorbance of samples/absorbance of controls × 100.

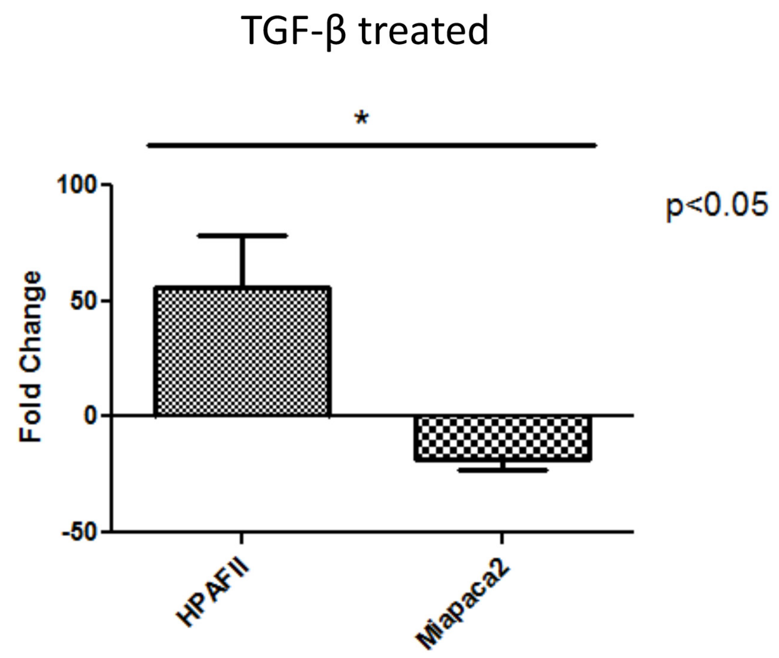

Supplementary Figure 4: HPAFII and MiaPaca2 were plated over transwell inserts pre-coated with reduced growth factor matrigel, and were allowed to invade the matrix towards serum contained in the bottom chamber for 24 hours. Percent invasion was calculated as absorbance of samples/absorbance of controls × 100.
